# Using three‐dimensional histology to visualize vascular tau accumulation in Alzheimer’s disease

**DOI:** 10.1002/alz.085808

**Published:** 2025-01-03

**Authors:** Zachary T Hoglund, Nancy E Ruiz‐Uribe, Eric del Sastre, Benjamin Woost, Joshua J Bailey, Theodore J Zwang, Rachel E Bennett

**Affiliations:** ^1^ Massachusetts General Hospital, Boston, MA USA; ^2^ Northeastern University, Boston, MA USA; ^3^ Massachusetts General Hospital, Charlestown, MA USA; ^4^ Harvard Medical School, Boston, MA USA; ^5^ Massachusetts Alzheimer’s Disease Research Center, Charlestown, MA USA

## Abstract

**Background:**

In cerebral amyloid angiopathy, amyloid beta accumulates within the walls of blood vessels and contributes to impaired vascular integrity and function. In this work, we observe that tau protein similarly builds up along blood vessels in Alzheimer’s disease brain.

**Method:**

We obtained frozen inferior temporal cortex from the Massachusetts Alzheimer’s Disease Research Center from n = 7 neuropathological confirmed Alzheimer’s disease donors and n = 6 normal aging controls. Three‐dimensional histology was carried out by cutting thick tissue sections (0.5 ‐ 1 mm) on a vibratome, clearing them using a modified CLARITY protocol, and immunolabeling for blood vessels, tau, neurons, and smooth muscle cells. Whole images were collected on a confocal or light sheet microscope and quantitatively examined using a combination of virtual reality tracing, MATLAB and Imaris. Adjacent tissues and isolated blood vessels were also measured for tau and phospho‐tau species using capillary western blotting to confirm imaging results.

**Result:**

In total, n = 107 Alzheimer’s disease and n = 127 control blood vessels were examined using three‐dimensional histology. Nearly all measured blood vessels in AD brain had regions of enhanced tau accumulation at the vascular surface (within 3 microns). Tau associated with blood vessels was found throughout cortical layers I‐V and was observed to be primarily located on arterioles. Further, isolated blood vessels were significantly enriched for the tau N‐terminus and phospho‐tau 181 and 217.

**Conclusion:**

These data indicate that tau accumulates on blood vessels in the Alzheimer’s disease brain to a greater extent than previously appreciated. Further, they highlight the utility of three‐dimensional histology methods for visualizing and quantifying novel relationships between features in the brain.

